# Chondroitin Sulfate-Based Nanocapsules as Nanocarriers for Drugs and Nutraceutical Supplements

**DOI:** 10.3390/ijms25115897

**Published:** 2024-05-28

**Authors:** Magdalena Górniewicz, Dawid Wnuk, Aleksander Foryś, Barbara Trzebicka, Marta Michalik, Mariusz Kepczynski

**Affiliations:** 1 Faculty of Chemistry, Jagiellonian University, Gronostajowa 2, 30-387 Kraków, Poland; magdalena.gorniewicz@doctoral.uj.edu.pl; 2 Doctoral School of Exact and Natural Sciences, Jagiellonian University, Prof. S. Łojasiewicza 11, 30-348 Krakow, Poland; 3 Department of Cell Biology, Faculty of Biochemistry, Biophysics and Biotechnology, Jagiellonian University, Gronostajowa 7, 30-387 Krakow, Poland; dawid.wnuk@uj.edu.pl (D.W.); marta.michalik@uj.edu.pl (M.M.); 4 Centre of Polymer and Carbon Materials, Polish Academy of Sciences, 41-819 Zabrze, Poland; aforys@cmpw-pan.pl (A.F.); btrzebicka@cmpw-pan.pl (B.T.)

**Keywords:** nanocapsules, chondroitin sulfate, bioactives, cytotoxicity, cryo-transmission electron microscopy

## Abstract

Oil-core nanocapsules (NCs, also known as nanoemulsions) are of great interest due to their application as efficient carriers of various lipophilic bioactives, such as drugs. Here, we reported for the first time the preparation and characterization of NCs consisting of chondroitin sulfate (CS)-based shells and liquid oil cores. For this purpose, two amphiphilic CS derivatives (AmCSs) were obtained by grafting the polysaccharide chain with octadecyl or oleyl groups. AmCS-based NCs were prepared by an ultrasound-assisted emulsification of an oil phase consisting of a mixture of triglyceride oil and vitamin E in a dispersion of AmCSs. Dynamic light scattering and cryo-transmission electron microscopy showed that the as-prepared core–shell NCs have typical diameters in the range of 30–250 nm and spherical morphology. Since CS is a strong polyanion, these particles have a very low surface potential, which promotes their stabilization. The cytotoxicity of the CS derivatives and CS-based NCs and their impact on cell proliferation were analyzed using human keratinocytes (HaCaTs) and primary human skin fibroblasts (HSFs). In vitro studies showed that AmCSs dispersed in an aqueous medium, exhibiting mild cytotoxicity against HaCaTs, while for HSFs, the harmful effect was observed only for the CS derivative with octadecyl side groups. However, the nanocapsules coated with AmCSs, especially those filled with vitamin E, show high biocompatibility with human skin cells. Due to their stability under physiological conditions, the high encapsulation efficiency of their hydrophobic compounds, and biocompatibility, AmCS-based NCs are promising carriers for the topical delivery of lipophilic bioactive compounds.

## 1. Introduction

Oil-core nanocapsules (NCs, also known as nanoemulsions) are versatile delivery systems for various lipophilic cargos [1,2]. They are composed of an inner oily core surrounded by polymer shells and can provide controlled drug release. Aqueous dispersions of such nanocapsules are formed by the emulsification of the oil phase with appropriate amphiphilic derivatives of charged polymers (e.g., polysaccharides such as hyaluronic acid) that form an envelope to stabilize the oil nanodroplets [3]. The advantage of such carriers is the great freedom in the choice of oil or mixtures of different oils making up the internal phase, and the wide range of polymers that can be used as the shell. A large part of bioactives is fat-soluble and tends to be susceptible to degradation [4]. Low water solubility, reduced chemical stability in the gastrointestinal tract, and susceptibility to rapid elimination from the body limit the bioavailability of bioactive substances, resulting in their low distribution in target tissues [5]. The use of carriers with an oil core solves the problem of the bioavailability of hydrophobic bioactives and protects them from harmful environmental conditions and reduces their toxicity. Such core–shell NCs have been used as carriers for active substances such as drugs or nutraceuticals, including oil-soluble vitamins (A, D, E, and K) [6], polyunsaturated fatty acids, e.g., omega-3 acids [7], and carotenoids [8], among others.

To date, hyaluronic acid (HA) or its amphiphilic derivatives have mostly been applied as polymer coatings to stabilize NCs [2,3,9]. HA belongs to glycosaminoglycans (GAGs) and is a weak polyanion, due to the fact that its structure contains only carboxyl groups whose dissociation depends on pH of the environment, which may affect the stability of HA-based NCs, depending on the medium. Chondroitin sulfate (CS), also belonging to glycosaminoglycans, is a strong polyanion, whose negative charge is due to the presence of carboxyl and sulfate groups being evenly distributed along the polysaccharide chain. The presence of sulfate groups provides this polysaccharide with a permanent negative charge, regardless of the pH of the environment. CS is one of the major structural components of animal cartilage, extracellular matrix (ECM), synovial fluid, and other animal connective tissues [10]. Due to the content and location of sulfate groups, there are several structural varieties of CS, which differ in their biological functions [11]. CS has well-recognized good pharmacological properties and is used in Europe as an anti-inflammatory drug [12]. In addition, CS, similar to HA, possesses a strong affinity for CD44 receptors [13], which are highly overexpressed in various types of cancer cells and determine the tumorigenic and metastatic capacities of cancer cells [14]. Therefore, CS can be used to prepare carriers that target cancer cells. CS was previously used to prepare oil-filled nanocapsules applied as carriers for doxorubicin [15] or vitamin E [1]. However, the formulation required the use of many additives in the form of emulgators or surfactants, such as hexadecyltrimethylammonium bromide (a cationic surfactant). In addition, unmodified CS is well soluble in water, and its deposition on the surface of oil droplets requires a positive charge at the water–oil interface (to allow for the electrostatic deposition of CS), which was achieved by introducing polycations such as polyethylenimine [15] or chitosan [16]. However, cationic polymers and surfactants are known for their strong cytotoxic properties. Modified CS, bearing hydrophobic side chains, may be an interesting alternative in the preparation of nanocapsules. Such polysaccharides are expected to adsorb spontaneously on the surface of oil droplets as a result of the penetration of their hydrophobic side groups into the oil phase, without the need for any cationic polymers or surfactants [9].

In this study, we describe the preparation of core–shell NCs coated with a CS-based membrane, without the use of any surfactants or polycations, which can be useful for the topical administration of actives. These nanocapsules consisted of an oily core and a shell composed of hydrophobically modified CS. We mainly focused on two objectives: (i) to determine the effect of the type of hydrophobic groups on the cytotoxicity of CS derivatives, and (ii) to determine the effect of the core oil on the stability of the nanoemulsion and the physicochemical and biological properties of the nanocapsules. To this end, we synthesized two amphiphilic CS derivatives (AmCSs) through covalent attachments of long alkyl groups, namely octadecyl (saturated) and oleyl (unsaturated) chains. Considering the potential applications of CS derivatives in pharmaceutical formulations and in various biomedical areas, we evaluated their biological properties. The cytotoxicity of the polymers against human skin fibroblasts (HSFs) and human keratinocytes (HaCaTs), which are the main types of cells that build the skin and are involved in wound healing processes, were determined and compared to the properties of the parent polymer. Then, using sonication, a series of NCs with a core containing a lipid/vitamin E (used as a model hydrophobic active) mixture and coated with AmCSs was prepared. The resulting nanocapsules were evaluated for their effects on the viability and proliferation of normal human skin cells. Our results are expected to be useful in understanding the effect of a polymer shell structure on the biological properties of the liquid-core nanocapsules.

## 2. Results and Discussion

### 2.1. Synthesis of the Amphiphilic CS Derivatives and Their Properties in Water

**Figure 1:**
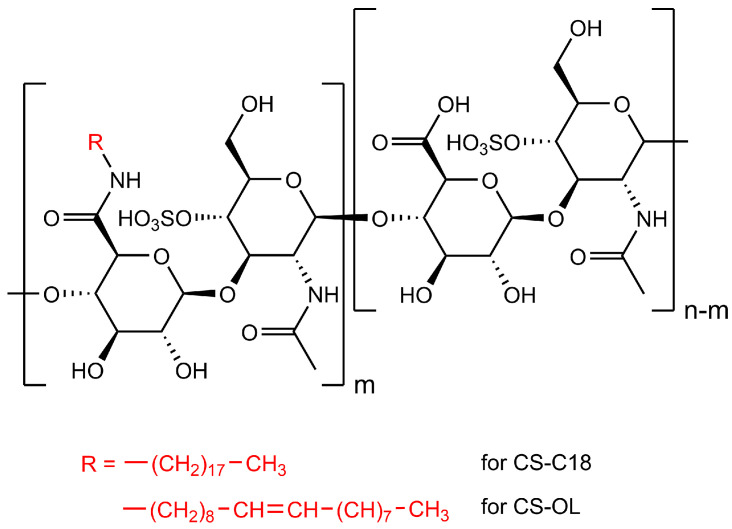
Chemical structures of hydrophobically modified CSs containing dodecyl (CS-C18) and oleyl (CS-OL) side groups.

CS was modified by covalent attachments of octadecyl (CS-C18) and or oleyl (CS-OL) groups along the polysaccharide chain (Figure 1). The CS derivatives were characterized by NMR and IR spectroscopy to confirm the structure and composition of the polymers and to determine the degree of substitution (DS) with the alkyl groups. The ^1^H NMR spectra of CS and its amphiphilic derivatives are shown in Figure S1. Additional peaks in the spectra of AmCSs at δ = 0.89 ppm and 1.26 ppm were assigned to the protons of the terminal methyl (–CH_3_) and the methylene (–CH_2_–) groups of the alkyl chains attached to CS, respectively. For CS-OL, a peak at ca. 5.4 ppm characteristic of the protons attached to carbons in double bonds (–CH=CH–) was also observed. In addition, an IR analysis of AmCSs was carried out. Figure S2 shows a comparison of FTIR spectra recorded for the native polysaccharide and its derivatives (CS-C18 and CS-OL, Figure 1). The spectra for the CS derivatives contained additional bands (i) in the range of 2930–2840 cm^−1^ characteristic of symmetric and asymmetric valence vibrations of the C-H bonds occurring in the CH_2_ and CH_3_ groups of the alkyl chains of the attached amines (IR spectra for pure oleylamine and octadecylamine are shown in Figure S3) and (ii) at about 1685 cm^−1^, corresponding to stretching vibrations of the C=O bonds in the secondary amides. Overall, the spectral analysis confirmed the successful attachment of the alkylamines to the carboxyl groups of CS.

The DS values were calculated based on the peak area ratio for the methyl protons of the CS acetamide groups and the methyl protons of the alkyl groups (see Section 3), and the results are listed in Table 1. The two derivatives had comparable DSs with the hydrocarbon groups. Importantly, the octadecyl and oleyl groups have the same length (18 carbons), but differ in the degree of unsaturation, which allowed us to study the effect of the presence of a double bond in the side groups on the properties of the AmCS-based NCs.

**Figure 2:**
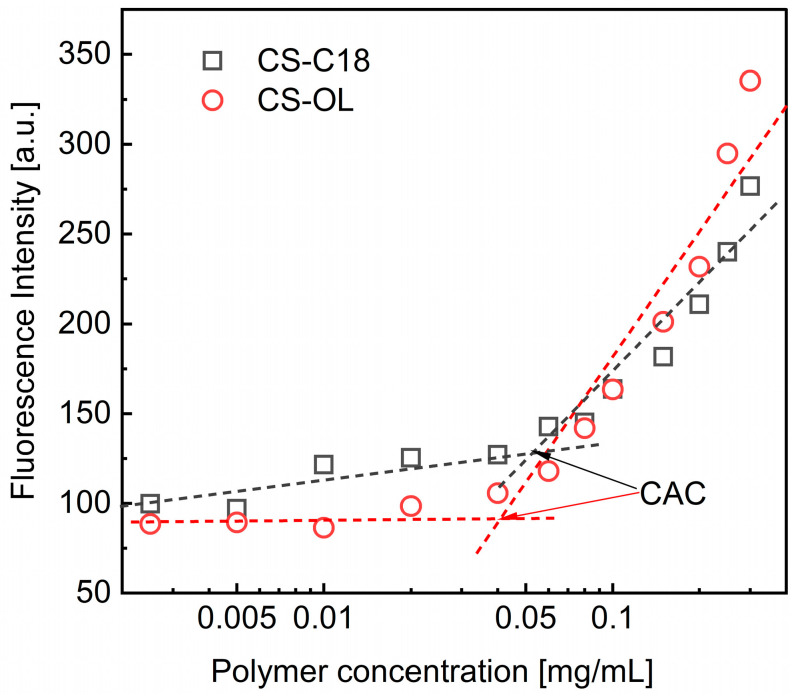
Dependence of fluorescence intensity of DPH (4 μM, λ_exc_ = 350 nm) on the logarithm of the CS-C18 and CS-OL concentration.

Amphiphilic derivatives of CS are assumed to self-organize into various structures when dispersed in aqueous media above certain concentrations (a critical aggregation concentration, CAC) [17]. Therefore, we first investigated the behavior of polymer dispersions in PBS. To determine the CAC, we followed changes in the fluorescence intensity of DPH, a molecular probe, as the concentration of amphiphilic CSs increased. The formation of polymer aggregates was manifested by a sharp increase in the fluorescence of the probe molecules as a result of their penetration into the forming hydrophobic domains by alkyl groups attached to CS derivatives (Figure 2). The measurements showed that the CACs for the amphiphilic CSs were in the range of tens of µg/mL (Table 1), which is consistent with previously published data [17,18,19]. For example, the CAC values for CSs grafted with α-tocopherol were 27–40 µg/mL, depending on the DS value (2.5–4.6%) [18]. Meanwhile, deoxycholic acid-substituted CS derivatives (2.5–7%) formed aggregates at concentrations of 27–50 µg/mL [19]. The larger CAC value measured for CS-C18 compared to CS-OL is due to the lower degree of substitution of the derivative with octadecyl groups. We previously showed that increasing CS grafting with alkyl groups leads to lower CAC values of amphiphilic derivatives [17].

The size of the structures formed by the AmCSs at a concentration of 1 mg/mL, exceeding the CAC point, was determined using the DLS method (Table 1). The polymers are self–assembled structures with hydrodynamic diameters in the range of 90–200 nm. The values of PI are relatively high, indicating a wide size distribution of objects suspended in the aqueous phase. The zeta potential of the CS-C18 and CS-OL nanostructures is highly negative, which is related to the presence of sulfate and carboxyl groups in the CS chemical structure. Our previous study using cryo-TEM and computer simulation indicated the formation of pseudo-micelles or loosely packed strongly hydrated nanogels in the dispersion of hydrophobically modified CS derivatives [17].

### 2.2. Formulation and Properties of Nanocapsules

**Figure 3:**
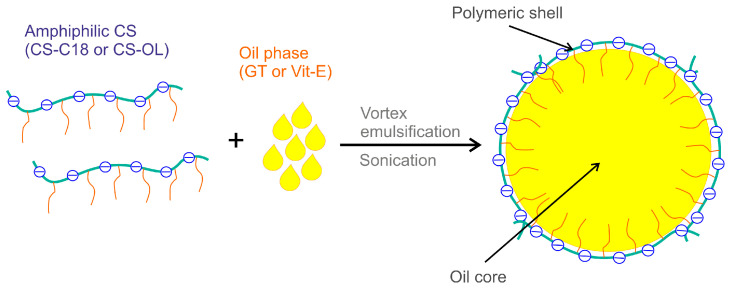
Preparation of oil-core nanocapsules stabilized by hydrophobically modified CS derivatives.

NCs coated with the CS derivatives (CS-C18 and CS-OL) with a core consisting of a GT/Vit-E mixture with different weight fractions of Vit-E: 0%, 20%, 50%, and 100% were obtained by mixing the oil phase and the polymer solution, and then sonicating the dispersion (Figure 3).

**Figure 4:**
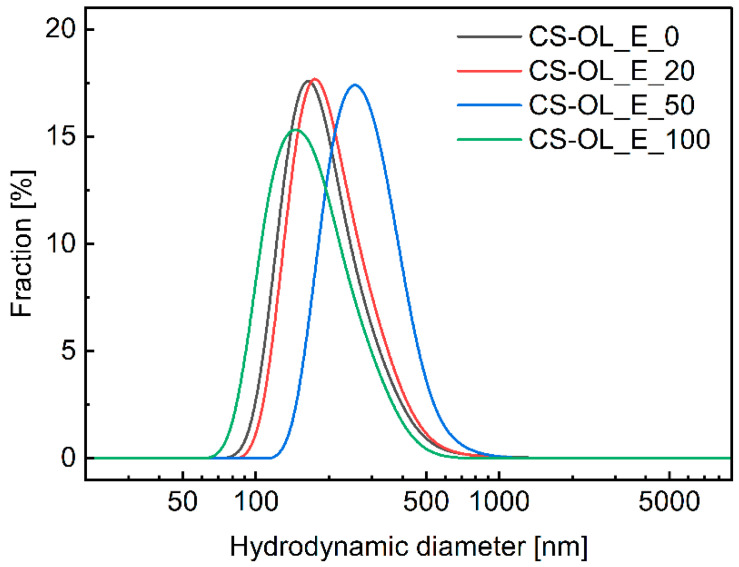
Number-weighted distribution profiles of hydrodynamic diameters measured by dynamic light scattering (DLS) for CS-OL-coated NCs with a core consisting of a GT/Vit-E mixture with different weight fractions of Vit-E: 0% (CS-OL_E_0), 20% (CS-OL_E_20), 50% (CS-OL_E_50), and 100% (CS-OL_E_100).

DLS and cryo-TEM were used to characterize the as-prepared core–shell NCs. Figure 4 shows the distribution profiles of the hydrodynamic diameters of the NCs determined by DLS measurements. The values of mean hydrodynamic diameter (*d*_z_) and polydispersity (PI) are collected in Table 2. The analysis of the DLS results indicates that there is no relationship between the core composition, the NC shell, and the sizes of the obtained nanoparticles. The average hydrodynamic diameters of oil nanodroplets were in the range of 300–600 nm. The PI values were relatively large, indicating a wide range in the sizes of the suspended phase. The distribution profiles for these emulsions remained almost constant over a period of several weeks.

In the next step, we measured the ζ-potential of the NCs. The change in surface charge of particles is generally mirrored by a change in the zeta potential (defined as the electric potential in the slip plane), whose values are well correlated with the stability of dispersions. Dispersions are assumed to be stable if the zeta potential is greater than +30 mV or less than −30 mV [20]. This ensures strong repulsive interactions between particles, which prevents their aggregation. The zeta potentials were determined using the microelectrophoretic method (Table 2). All the formulations were characterized by negative zeta potential values, indicating that the amphiphilic polysaccharides can adsorb at the water–oil interface, with alkyl groups penetrating into the oil phase. Interestingly, the lowest ζ values were measured for NCs with pure GT or Vit-E cores. For mixed-core systems, an increase in the zeta potential was observed, and this is particularly evident for the 1:1 GT/Vit-E mixture. This is probably related to the limited miscibility of Vit-E with triglyceride oil in the weight ratios used, which may result in areas where vitamin E accumulates in larger amounts, thus affecting changes in adsorption at the water–oil interface and the conformation of amphiphilic chains of CS derivatives. However, the exact reasoning behind the observed changes in zeta potential with the composition of the oil core requires further research. Due to the unusual zeta changes, systems containing only pure oil cores were selected for further study (CS-C18_E_0, CS-C18_E_100, CS-OL_E_0, and CS-OL_E_100).

**Figure 5:**
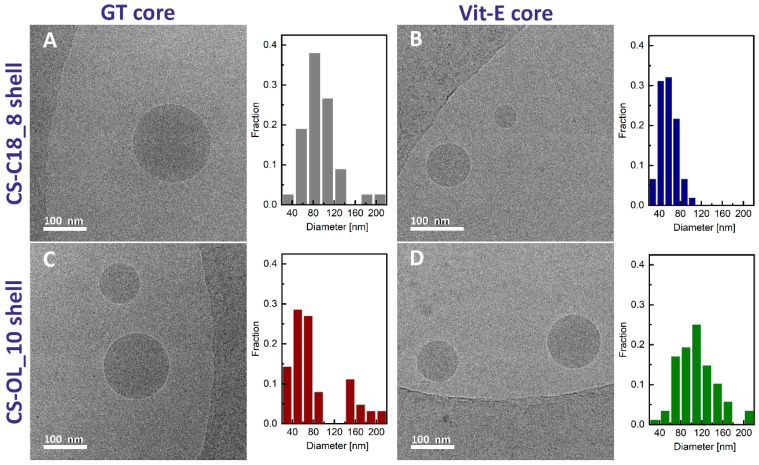
Cryo-TEM micrographs of the dispersion of GT (**A**) and Vit-E (**B**) in CS-C18 solution (CS-C18_E_0 and CS-C18_E_100, respectively) and the dispersion of GT (**C**) and Vit-E (**D**) in CS-OL solution (CS-OL_E_0 and CS-OL_E_100, respectively). The corresponding diameter profiles of the nanoparticles are shown on the right.

We used cryo-TEM microscopy to visualize the morphology of the nanocapsules with pure GT or Vit-E cores and covered with CS-C18 or CS-OL. Figure 5 shows typical cryo-TEM micrographs for the dispersions and the corresponding diameter distributions. The images reveal the presence of particles of a good spherical shape and diameters mostly in the range of 30–250 nm. These values are lower than that estimated using the DLS measurements. This can be explained by the fact that the DLS method measures the z-average mean hydrodynamic diameter, which is heavily biased towards the largest structures in the dispersion.

### 2.3. Intracellular Uptake of the Nanocapsules

**Figure 6:**
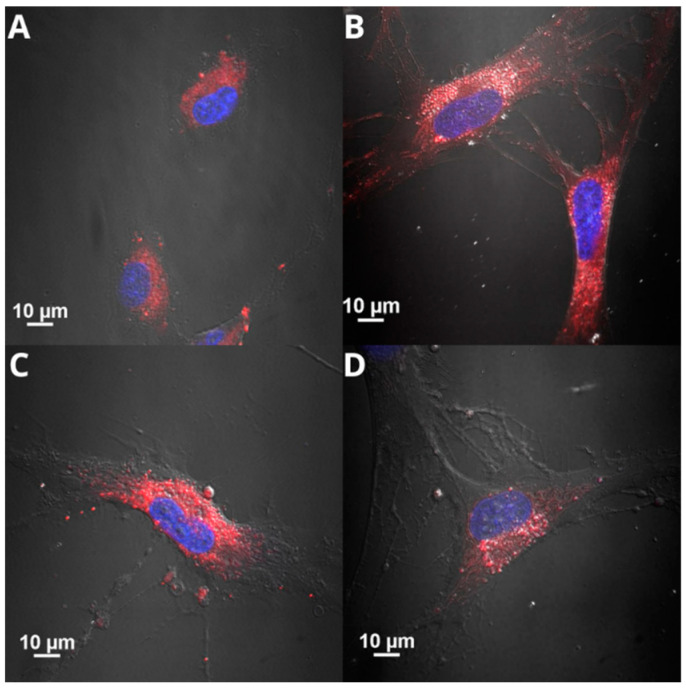

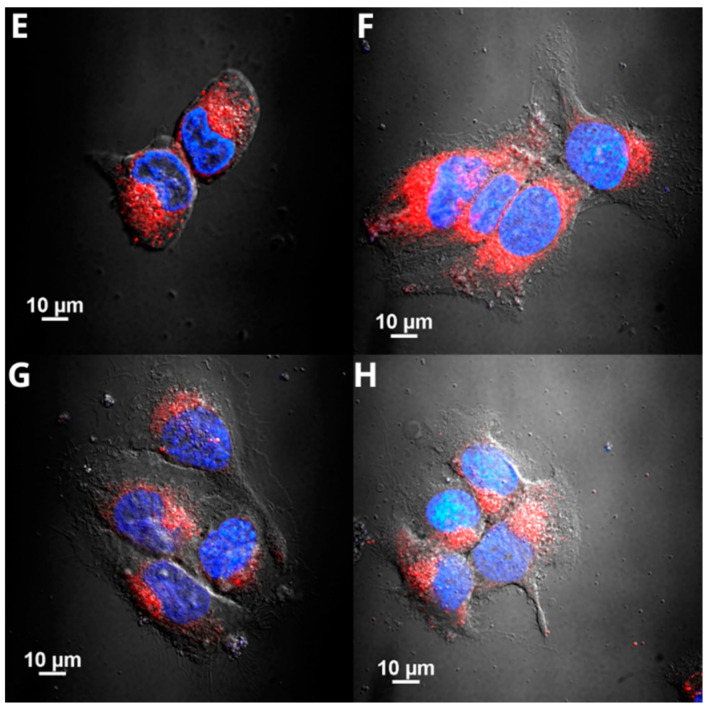
Internalization of fluorescently stained (red fluorescence) nanocapsules by HSFs ((**A**), CS-C18_E_0; (**B**), CS-C18_E_100; (**C**), CS-OL_E_0; (**D**), CS-OL_E_100) and HaCaTs ((**E**), CS-C18_E_0; (**F**), CS-C18_E_100; (**G**), CS-OL_E_0; and (**H**), CS-OL_E_100)) observed using CLSM microscopy after 1-day incubation. The nuclei were stained with Hoechst 33342 (blue fluorescence).

To assess the intracellular uptake of AmCS-coated nanocapsules by HSFs and HaCaTs, their oily core was stained with NR. The cells were incubated with fluorescently labeled NCs for 1 day and imaged by CLSM microscopy (Figure 6). The cells exposed to NR-loaded NCs showed red fluorescence (characteristic of NR) at the cytoplasmic level (Figure 6). The contours of the cells were determined by transmitted light imaging, and Hoechst dye was used to stain the cell nuclei (blue fluorescence). The micrographs reveal that the fluorescence emitted by the NR-stained NCs is located inside the cells. In addition, three-dimensional reconstructions of fluorescence micrographs and cell cross-sections (Figure S4) indicate that the red fluorescence extends through the entire cell volume. Thus, it can be assumed that NCs enter the cell and localize near the cell nuclei. Based on these observations, it can be concluded that the nanocapsules were effectively internalized by both cell types.

### 2.4. Effects of Polymer Dispersions and CS-Based Nanocapsules on Proliferation and Viability of Human Skin Cells

The cytotoxicity of polymers is a very important issue for their use in biotechnological and biomedical applications [21]. We investigated the effects of AmCS dispersions and nanocapsules on the proliferation and viability of human skin cells in culture. Cytotoxicity and proliferation assays were conducted on primary human skin fibroblasts (HSFs) and human keratinocytes (HaCaT cell line, a pivotal model in dermatological research) in an in vitro culture setting. Both cell types play a key role in regulating skin physiology and healing skin wounds. The cell viability and proliferation were assessed using MTT (assesses the mitochondrial dehydrogenase activity of cells) and CV tests [22], respectively, after 5-day incubation with the polysaccharides or their nanocapsules. The CV test is a simple method for determining the relative number of adherent cells in a culture by staining them with crystal violet, which binds primarily to DNA [23].

**Figure 7:**
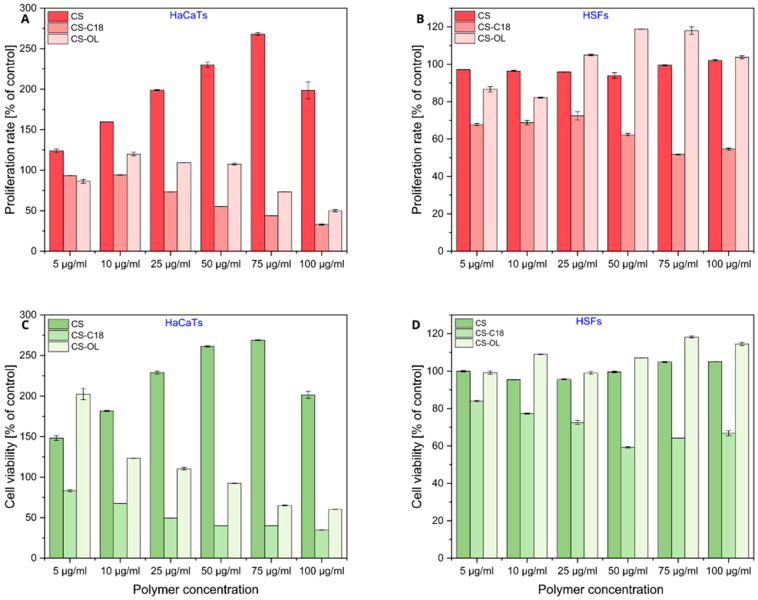
Effect of the amphiphilic CSs on human skin cell proliferation and viability. HaCaTs (**A**,**C**) and HSFs (**B**,**D**) were treated with increasing concentrations (5–100 µg/mL) of CS and two amphiphilic derivatives of CS (CS-C18 and CS-OL) for 5 days. The proliferation rate (**A**,**B**) and the viability (**C**,**D**) of the cells were determined using the CV and MTT assays. The results are presented as a percentage of control cells cultured in medium without amphiphilic CS for 5 days. The results are average values from three and two independent experiments for MTT and CV assess, respectively. The error bars are the standard deviations.

Figure 7 shows the proliferation of HSFs and HaCaTs after 5 days of incubation with different concentrations of the parent polysaccharide and its amphiphilic derivatives. The unmodified CS showed no cytotoxicity against either cell type. In addition, the results revealed a pronounced pro-proliferative effect of CS on HaCaTs, which was concentration-dependent. Both the MTT assay and the CV assay indicate a significant increase in the number of cells in samples incubated for 5 days with CS, with viability increasing significantly with increasing polysaccharide concentration. At a polymer concentration of 75 µg/mL, the number of viable cells exceeds 2.5 times that of the control, underscoring the positive impact of CS on the growth and division of HaCaTs. In contrast, for HSFs, we observed only a slight improvement in cell proliferation at higher concentrations of CS in the medium, suggesting that the effect of CS on cell proliferation varies by cell type. Our findings are consistent with the previous reports. For example, Katayama et al. showed that CS strongly stimulated the proliferation of keloid-derived fibroblasts, but not normal dermal fibroblasts [24]. It has also been reported that CS enhances the proliferation of other human cells, such as normal human keratinocytes and dermal fibroblasts [25], the HaCaT cell line and mesenchymal stem cells (hMSCs) [26], and human chondrocytes [27]. In turn, Zou et al. demonstrated that the biological effects of chondroitin sulfate appear to be dependent on the presence and position of the sulfate groups [28]. They showed that treatment with chondroitin 6-sulfate caused a dose-dependent increase in the adhesion and proliferation of rabbit palate fibroblasts, while exposure to chondroitin 4-sulfate resulted in decreased cell adhesion.

In the case of amphiphilic CS derivatives, their cytotoxic effect strongly depended on the cell type. CS-C18 showed limited but concentration-dependent toxicity to HSFs. The results of the two assays coincide and show that the number of viable cells after 5-day incubation in the presence of this polymer at concentrations of 50–100 µg/mL dropped to about 60% of that in the control. On the contrary, CS-OL was completely nontoxic to HSFs. More importantly, at concentrations above 25 µg/mL, this polymer increased cell proliferation. The effect of both polymers on the viability of keratinocytes was more pronounced. A substantial increase in cytotoxicity was observed with increasing polymer concentration. The determined IC_50_ values were 62 µg/mL and 97 µg/mL for CS-C18 and CS-OL, respectively, indicating that, as in the case of HSFs, the derivative with C18 groups showed greater cytotoxicity compared to CS-OL. The cytotoxicity of CS and CS-C18 against mesenchymal stem cells has been studied previously [29]. It was shown that while CS is completely non-toxic, the amphiphilic polymer reduces cell viability in a DS-dependent manner. This is consistent with our current observations that the chemical modification of CS can lead to a reduction in CS biocompatibility. However, the chemical structure of the hydrophobic groups used for the modification has a significant effect on the observed activity of the amphiphilic polysaccharide against cells.

**Figure 8:**
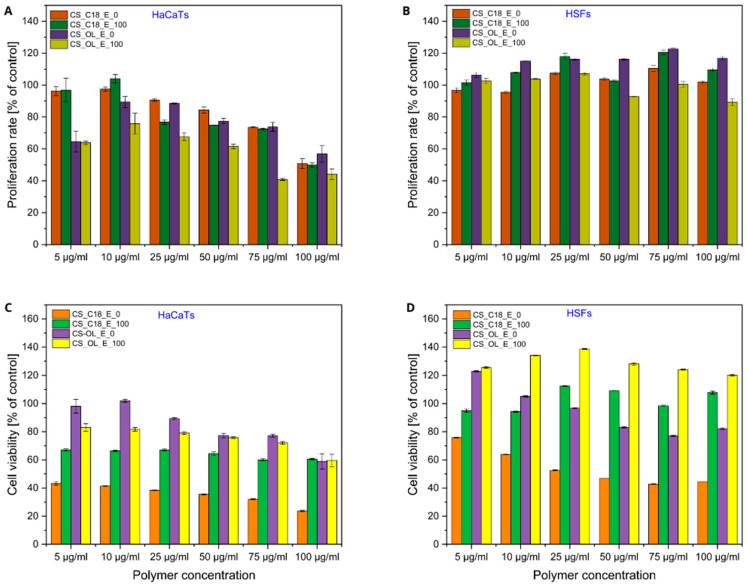
Effect of the AmCS-NCs with the GT and Vit-E core on the proliferation (**A**,**B**) and viability (**C**,**D**) of HaCaTs (**A**,**C**) and HSFs (**B**,**D**). The cells were exposed to increasing concentrations (5–100 µg/mL) of nanocapsules with pure GT (CS_C18_E_0 and CS_OL_E_0) or Vit-E (CS_C18_E_100 and CS_OL_E_100) cores for 5 days. The proliferation rate (**A**,**B**) and the viability (**C**,**D**) of the cells were determined using the CV and MTT assays. The results were presented as a percentage of control cells cultured in medium without CS-NCs for 5 days. The results are an average value from three and two independent experiments for MTT and CV assess, respectively. The error bars are the standard deviations.

Our further studies focused on the effect of AmCS-based NCs on HaCaTs and HSFs. Cell proliferation and viability were evaluated in a medium comprising increasing concentrations of NCs with a core containing GT or Vit-E and coated with CS-C18 or CS-OL. Figure 8 shows the results of MTT and CV assays performed for cells treated with the AmCS-based NCs. As shown by the CV assay results, a 5-day culture of HSFs in media containing the tested formulations did not cause significant changes in cell proliferation in the range of polymer concentrations from 5 to 100 µg/mL. In contrast, a slight effect of NCs on HaCaT proliferation was observed over the concentration range tested. The nanocapsules at concentrations up to 25 μg/mL in the medium showed no significant effect on the number of cells compared to that under standard culture conditions. However, a further increase in the concentration of these NCs in the culture medium resulted in a slight concentration-dependent decrease in keratinocyte proliferation, indicating the varying effects of AmCS-based NCs on different human skin cell types (Figure 8A,B).

To determine whether the observed effects of the tested formulations on HSF and HaCaT cell proliferation correlated with cytotoxicity, cell viability analyses were performed using the MTT assay (Figure 8C,D). The presence of CS-C18_E_0 NCs in the medium resulted in the significant inhibition of HSF mitochondrial activity in a dose-dependent manner. Cell viability at the highest content of these NCs was about 40–50% of that of cells in the control sample. The effect of these nanocapsules on HaCaTs was similar, but more pronounced. In contrast, CS-OL_E_0 NCs had much less of an effect on the metabolic activity of HSFs and HaCaTs, observed only at nanoparticle concentrations above 25 µg/mL. Our results showed that the use of amphiphilic CS derivatives as nanocapsule shells altered their cytotoxic effects on skin cells compared to the corresponding CS derivatives in dispersion. However, the observed effect strongly depended on the type of coating polymer and the type of cells.

Interestingly, both types of NCs with a Vit-E-filled core showed a preventive effect on skin cell viability compared to those with a triglyceride core. Vitamin E was previously reported to protect human epidermal keratinocytes [30] and human skin fibroblasts [31] against a UV-induced decrease in viability or oxidative stress conditions. A similar role of vitamin E on skin cell survival was observed by Butt et al., who demonstrated the cytoprotective effects of vitamin E on both human skin fibroblasts and human epidermal keratinocytes against thermal injury [32,33]. Our results indicate that vitamin E, a hydrophobic bioactive substance, administered to the culture medium in nanocapsules coated with AmCSs, also had cytoprotective effects on human skin cells. However, the observed effect depended on the type of cells and the coating polymer. Our findings suggest the potential use of AmCS-based NCs as carriers of lipophilic bioactive compounds.

## 3. Materials and Methods

### 3.1. Materials

Chondroitin sulfate A sodium salt (CS) from bovine trachea was purchased from Acros Organics. *N*-(3-dimethylaminopropyl)-*N*′-ethylcarbodiimide hydrochloride (EDC), *N*-hydroxysuccinimide (NHS, 98%), 1,6-diphenyl-1,3,5-hexatriene (DPH), phosphate-buffered saline (PBS, tablets), octadecylamine (≥99%, GC), oleylamine (technical grade, 70%) and dialysis tubing cellulose membrane (typical molecular weight cut-off 14,000 Da), glyceryl trioctanoate (GT), (±)-α-tocopherol (Vit-E, synthetic, ≥96% HPLC), and nile red (NR, for microscopy) were obtained from Sigma-Aldrich. Formamide (pure p.a.), N,N-dimethylformamide (DMF), and ethanol were obtained from Chempur. Ultrapure Milli-Q water was used in the experiments.

### 3.2. Synthesis of Amphiphilic CS Derivatives

The covalent attachment of alkyl groups to CS chains was performed according to the previously reported procedure [17]. Briefly, CS (500 mg) was dissolved in formamide (100 mL) at 60 °C and cooled down to room temperature. EDC (187.68 mg) and NHS (312.63 mg) were added to the CS solution. The mixture was stirred for 20 min at room temperature to activate the carboxyl groups of CS. Amine (29 mg) dissolved in DMF (20 mL) was then slowly added to the CS solution. The reaction mixture was stirred for 20 h at room temperature. To remove organic solvents and purify the product from unreacted amine and byproducts, the mixture was dialyzed against ethanol and mixtures of ethanol and PBS with gradually increasing amounts of PBS (ethanol/PBS 4:1, 2:1, 1:1, 1:2 by volume), followed by pure PBS and water, for 1 day in each medium. Amphiphilic CS derivatives were recovered by lyophilization and characterized by IR and ^1^H-NMR spectroscopy to confirm their structure and determine the DS. FTIR spectra were recorded using a Thermo Scientific Nicolet iS10 FT-IR spectrometer with ATR accessory (Figures S2 and S3). NMR spectra taken using a Bruker Avance III HD 400 MHz NMR spectrometer at 80 °C are shown in Figure S1.

To calculate degree of substitution (DS) values for the AmCSs, we used the peaks at 0.89 ppm and 2.0 ppm assigned to the methyl protons of the oleyl or octadecyl side groups (–CH_2_CH_2_CH_3_) and the methyl protons of the acetamide group of CS (–NH(C=O)CH_3_), respectively. These peaks are labeled in Figure S1 as peak A and B. Both signals correspond to three protons, so the DS was calculated as the ratio of peak area at 0.89 ppm to peak area at 2.0 ppm.

### 3.3. Preparation of Nanocapsules

AmCS-coated NCs were obtained by the sonication-assisted emulsification of the oil phase in a solution of the amphiphilic polysaccharides (Figure 3). Solutions of the amphiphilic CS derivatives with a concentration of 1 mg/mL were prepared. A 1 mM PBS solution filtered through a 22 μm filter was used as a solvent. The solutions were left overnight on a magnetic stirrer at 100 rpm. The GT oil or its mixture with α-tocopherol (a total weight of 10 mg) was added to the polymer solution at an oil/water phase volume ratio of about 1:200. The samples were shaken using a vortex shaker (IKA, Königswinter, Germany) for 5 min and then sonicated for 2 min using a VCX 500 sonicator (Sonics & Materials. Inc., Newtown, CT, USA) (power—150 W, pulse duration—1 s, break duration—1 s). Mixtures of GT and α-tocopherol with weight ratios of 10:0, 4:1, 1:1, and 0:10 (*w*/*w*) were used in the study. Cells stained with nile red (NR) were prepared by adding 0.01 mg of NR dissolved in ethanol to the nanocapsule suspension.

### 3.4. Critical Aggregation Concentrations (CACs)

The CAC of AmCSs was determined using the previously reported procedure [34]. Briefly, a series of samples containing the polymer at concentrations ranging from 0 to 0.1 mg/mL and DPH at a constant concentration (4 μM) were prepared and stirred for 1 h in the dark. The fluorescence spectra of the samples were measured using a Hitachi F-7000 spectrofluorometer (λ_ex_ = 350 nm). The CAC values were determined from the dependence of the fluorescence intensity on the polymer concentration, as the intersection of two lines fitted for low polymer concentrations and for concentrations at which the DPH fluorescence intensity increased steeply.

### 3.5. Dynamic Light Scattering (DLS) and Zeta Potential Measurements

DLS and ζ-potential measurements were performed using a Zetasizer Ultra apparatus (Malvern Instrument Ltd., Malvern, UK) recording the light scattered by the sample at a backscatter angle of 173°. The measurements were performed for concentrations significantly exceeding the experimentally determined CAC and were carried out at 25 °C in PBS buffer (pH 7.4). The data represent the average result of two independent experiments. The hydrodynamic mean diameter (*d*_z_), dispersity index (PI), and distribution profile of each sample were calculated using ZS Xplorer v3.2.0 software provided by the manufacturer.

### 3.6. Cryo-TEM

Cryogenic Transmission Electron Microscopy (cryo-TEM) images were obtained using a Tecnai F20 X TWIN microscope (FEI Company, Hillsboro, OR, USA) equipped with a field emission gun, operating at an acceleration voltage of 200 kV. Images were recorded on a Gatan Rio 16 CMOS 4k camera (Gatan Inc., Pleasanton, CA, USA) and processed with Gatan Microscopy Suite (GMS, 3.31.2360.0) software (Gatan Inc., Pleasanton, CA, USA). Specimen preparation was performed by the vitrification of the aqueous solutions on grids with holey carbon film (Quantifoil R 2/2; Quantifoil Micro Tools GmbH, Großlöbichau, Germany). Prior to use, the grids were activated for 15 s in oxygen plasma using a Femto plasma cleaner (Diener Electronic, Ebhausen, Germany). Cryo-samples were prepared by applying a droplet (3 μL) of the suspension to the grid, blotting with filter paper and immediately freezing in liquid ethane using a fully automated blotting device Vitrobot Mark IV (Thermo Fisher Scientific, Waltham, MA, USA). After preparation, the vitrified specimens were kept under liquid nitrogen until they were inserted into a cryo-TEM-holder Gatan 626 (Gatan Inc., Pleasanton, CA, USA) and analyzed in the TEM at −178 °C.

### 3.7. Cytotoxicity and Proliferation Assays

Human keratinocytes (HaCaTs) were obtained from CLS (Cell Lines Service GmbH, Eppelheim, Germany). HaCaTs and primary human skin fibroblasts (HSFs, ATCC, and PCS-201-012) were cultured in Dulbecco’s Modified Eagle Medium (DMEM; Sigma-Aldrich; Cat. D6429, St. Louis, MO, USA) supplemented with 10% fetal bovine serum (FBS, Gibco, Cat: A5256701, New York, NY, USA) and an antibiotic–antimycotic cocktail (antibiotic–antimycotic 100×; Gibco; Cat.: 15240062) under standard conditions: 5% CO_2_, 37 °C, and 95% air humidity in a cell culture incubator. The cells were passaged at 85–90% confluence using 0.05% trypsin solution in PBS without Ca^2+^ and Mg^2+^ ions, cultured in Falcon culture flasks, and then seeded for experiments in 96-well plates (Falcon) at a density of 5 × 10^3^ cells per cm^2^. The cells were incubated in the culture medium for 24 h, and then the medium was changed to serum-free conditions in DMEM with the addition of 0.1% bovine serum albumin (BSA, Sigma-Aldrich, Cat: A9418). After 24 h of incubation, the cells were stimulated with CS or its derivatives (CS-C18 and CS-OL) at concentrations of 5, 10, 25, 50, 75, or 100 μg/mL dissolved in PBS with Ca^2+^ and Mg^2+^ ions, and incubated for 1, 3, or 5 days. After this time, a 2-h incubation of the cells with sterile 3-(4,5-dimethyl-2-thiazolyl)-2,5-diphenyl-2H-tetrazolium bromide (MTT, Sigma-Aldrich, Cat: M5655; 5 mg/mL in PBS with Ca^2+^ and Mg^2+^ ions) was performed, the medium was aspirated, and the formazan crystals were dissolved in isopropanol (Sigma-Aldrich, Cat: 109827). A cell proliferation assay with crystal violet (CV) [35] was performed under the same conditions as the MTT assay. After incubation with CS or its derivatives, the cells were fixed with formaldehyde (4%) and incubated for 15 min with a CV solution (5% *w*/*v* dissolved in 1:4 methanol/water). After incubation, the solution was removed, and the cells were rinsed 5 times with deionized water and dried. Then, 100 µL of solubilizing solution (13% *w*/*v* citric acid; 10% *w*/*v* trisodium citrate dissolved in 1:1 methanol/water) was added per well and incubated with the cells (30 min) until the dye encapsulated inside the cells was dissolved. The assays were followed by absorbance measurements using a microplate reader (Multiskan FC, Thermo Fisher Scientific, Waltham, MA, USA) at 570 nm (for the MTT assay) and 540 nm (for the CV assay). The results were presented as percentages relative to the control without added compounds. Three and two independent experiments were performed for MTT and CV assays, respectively. The half-maximal inhibitory concentration (IC50) was determined as the polymer concentration at which a 50% reduction in cell growth was observed compared to the control sample.

## 4. Conclusions

Nanocapsules containing cores of a mixture of triglyceride oil and vitamin E (used as a hydrophobic bioactive substance) coated with amphiphilic CS derivatives were fabricated by ultrasound-assisted emulsification to study the effects of the core composition and the structure of the shell-forming polymer on the physicochemical and biological properties of the obtained NCs. The as-prepared NCs were spherical in shape and 30–250 nm in diameter. The deposition of CS, which is a strong polyanion with sulfate groups, on the surface of oil nanodroplets provided them with a low surface potential, which promotes the stabilization of the nanoemulsion through strong repulsive electrostatic interactions. The alkyl groups of CS derivatives penetrate the oil phase, while the hydrophilic polysaccharide chains with anionic groups remain in the aqueous phase, preventing aggregation and fusion processes. Concurrently, we observed a previously unseen effect of the oil core composition on the surface potential of NCs, which is probably due to the limited solubility of the oil phase components.

In vitro studies on HSFs and HaCaTs showed that although the parent polymer is non-toxic to normal skin cells and, in the case of keratinocytes, strongly enhances their proliferation, its amphiphilic derivatives can exhibit mild cytotoxic properties, especially against keratinocytes over multi-day incubation. However, the magnitude of the negative effect on cells strongly depended on the type of side groups used to modify CS. The selection of unsaturated alkyl groups instead of saturated ones for CS modification can substantially improve the biocompatibility of the derivatives. In addition, the use of the amphiphilic polysaccharides as nanocapsule coatings reduces their harmful effects on the viability and proliferation studies of normal skin cells. In summary, the promising results obtained for AmCS-based NCs indicate their potential use in dermatology and cosmetics as carriers of lipophilic bioactive compounds applied directly to the skin in formulations such as ointments and creams.

## Figures and Tables

**Table 1:** Values of the degree of substitution (DS) and critical aggregation concentration (CAC) for the amphiphilic CSs. Values of the mean hydrodynamic diameter (*d*_z_), polydispersity (PI), and zeta potential (ζ) measured for dispersions of the amphiphilic CSs (1 mg/mL) in 1 mM PBS. Values are the mean ± standard deviation, *n* = 3.

Polymer	DS [%]	CAC [µg/mL]	*d*_z_ [nm]	PI	ζ [mV]
CS-C18	8	53.1	202 ± 71	0.36 ± 0.01	−33.3 ± 0.3
CS-OL	10.5	40.0	95 ±21	0.50 ± 0.05	−36.8 ± 1.7

**Table 2:** Values of the mean hydrodynamic diameter (*d*_z_), polydispersity (PI), relative standard deviation (RSD), and zeta potential (ζ) of CS-coated NCs dispersed in a 1 mM PBS solution. Values are the mean ± standard deviation, *n* = 3.

Sample	Weight of GT [mg]	Weight of Vit-E [mg]	*d*_z_ [nm]	PI	RSD [%]	ζ [mV]
CS-C18_E_0	10	0	315.7 ± 5.7	0.34 ± 0.02	1.8	−24.6 ± 2.1
CS-C18_E_20	8	2 (20%)	304.7 ± 6.0	0.22 ± 0.02	2.0	−24.5 ± 0.7
CS-C18_E_50	5	5 (50%)	391.5 ± 2.5	0.38 ± 0.03	0.6	−18.1 ± 1.8
CS-C18_E_100	0	10 (100%)	406 ± 13	0.37 ± 0.02	3.2	−31.8 ± 0.9
CS-OL_E_0	10	0	390.4 ± 0.5	0.33 ± 0.04	0.1	−30.4 ± 1.7
CS-OL_ E_20	8	2 (20%)	366.1 ± 3.5	0.27 ± 0.02	0.9	−21.6 ± 2.1
CS-OL_E_50	5	5 (50%)	571 ± 29	0.42 ± 0.05	5.0	−15.0 ± 2.5
CS-OL_E_100	0	10 (100%)	371 ± 15	0.47 ± 0.07	4.0	−31.6 ± 0.3

## Data Availability

Data are contained within the article and the Supplementary Materials.

## Supplementary Materials

The following supporting information can be downloaded at https://www.mdpi.com/article/10.3390/ijms25115897/s1.
