# Supplementary material for: Chondroitin Sulfate-Based Nanocapsules as Nanocarriers for Drugs and Nutraceutical Supplements
Source: Int J Mol Sci. 2024 May 28;25(11):5897. doi: 10.3390/ijms25115897 (PMC11172538; doi:10.3390/ijms25115897)
Supplement: Supplementary file 1 [file ijms-25-05897-s001.zip › ijms-2984342-supplementary.pdf]

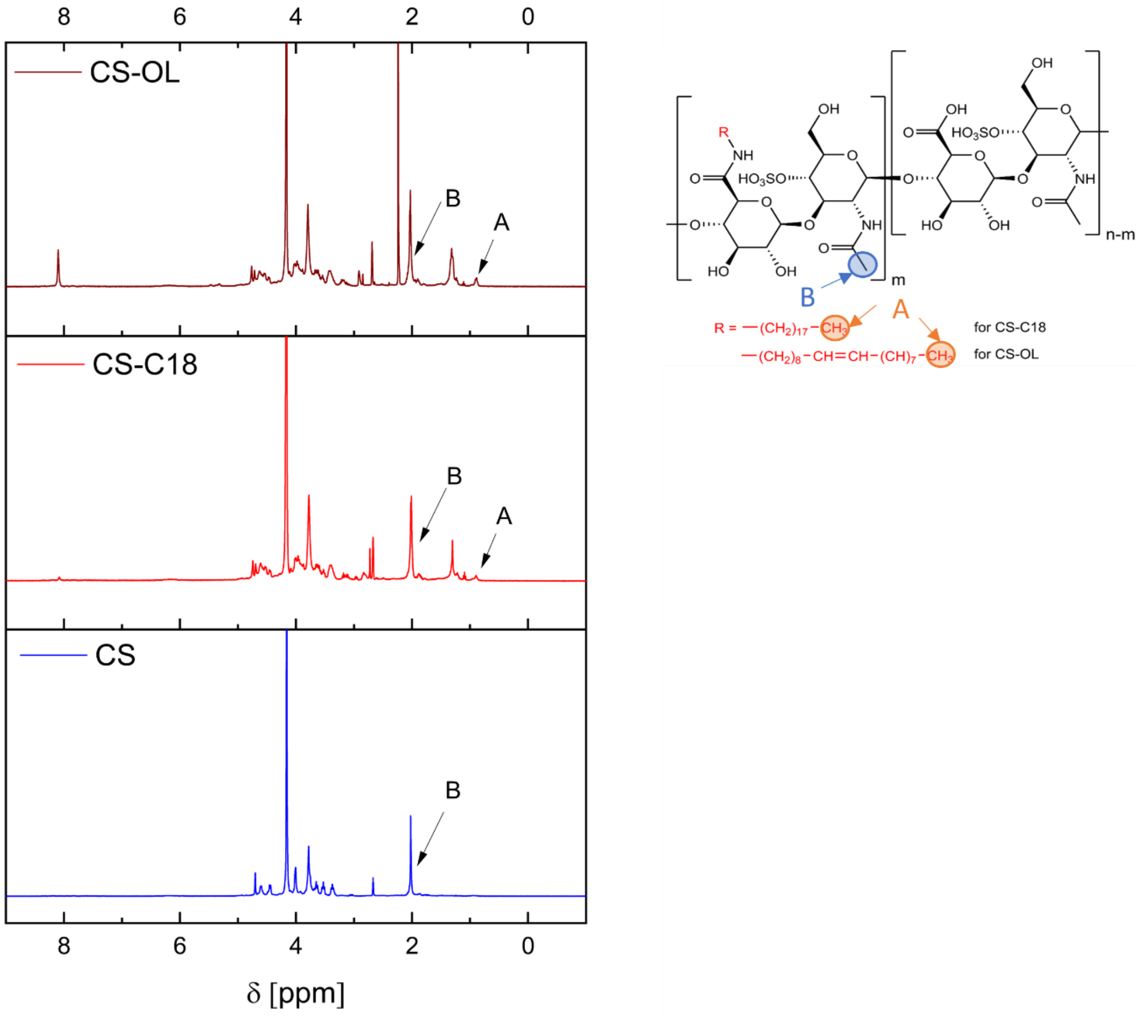

Figure S1. ^{1}H NMR spectra of CS, CS-C18, and CS-OL dissolved in the D_2O/DMSO-d_6 mixture (4:1, v/v) at 80°C.

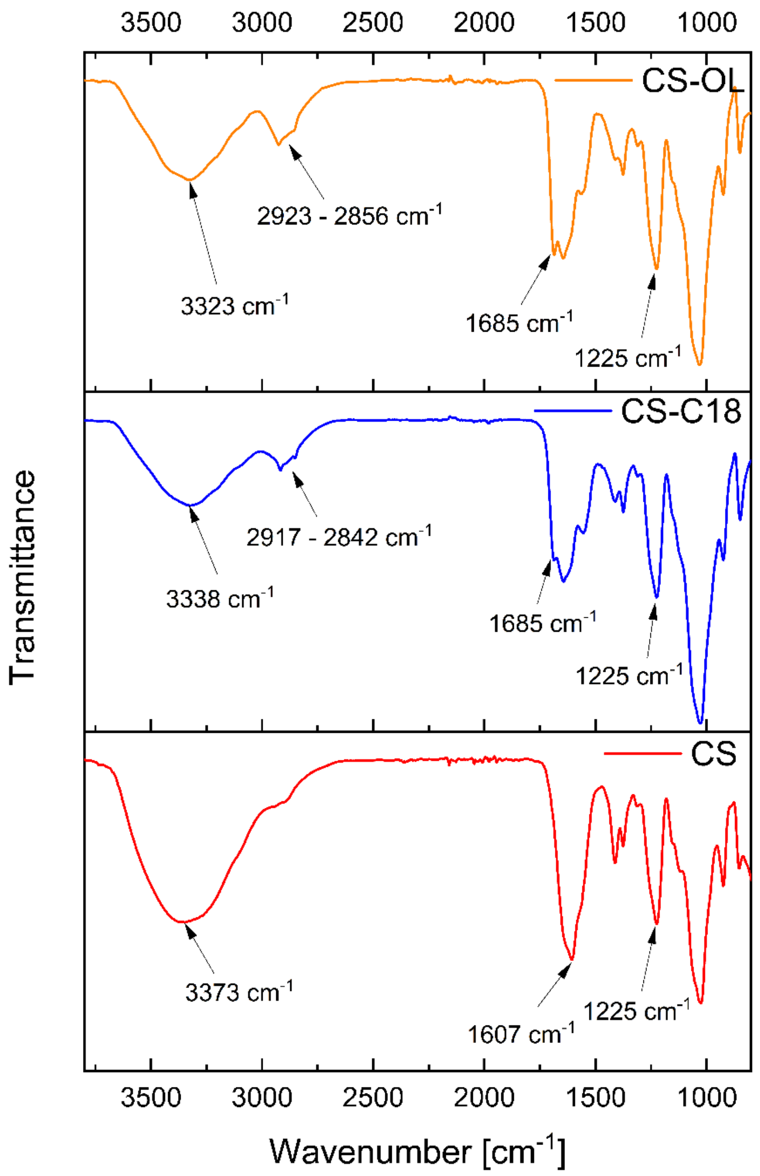

Figure S2. ATR-FTIR spectra of CS, CS-C18, and CS-OL.

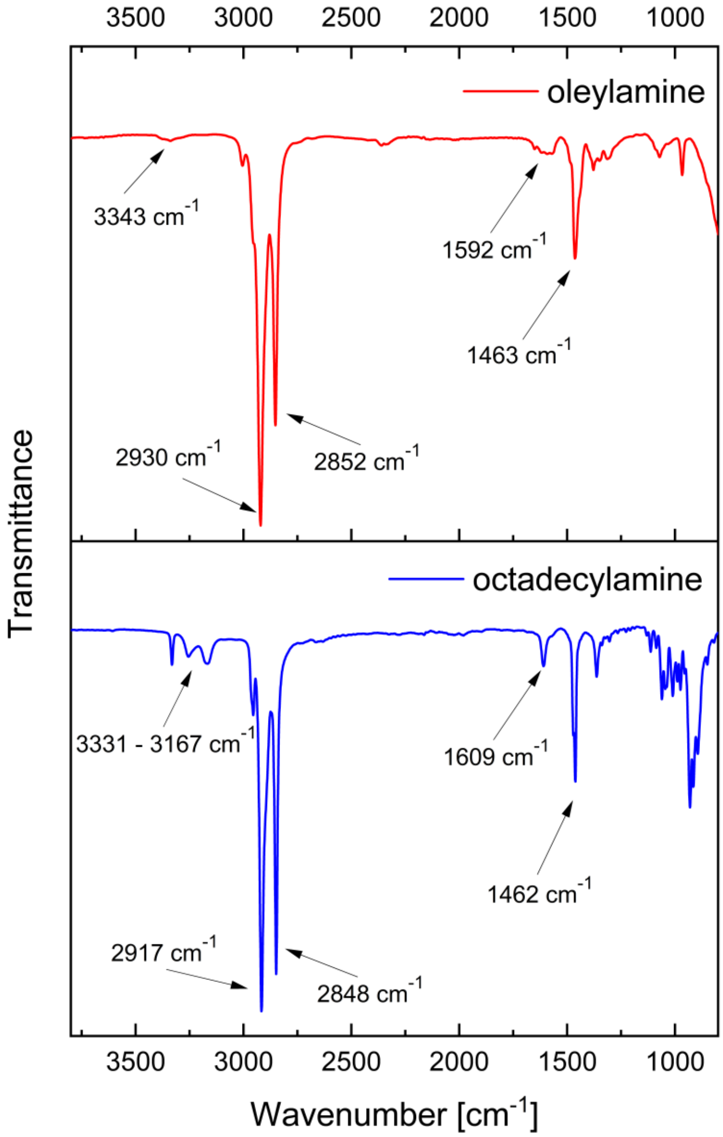

Figure S3. ATR-FTIR spectra of oleylamine and octadecylamine.

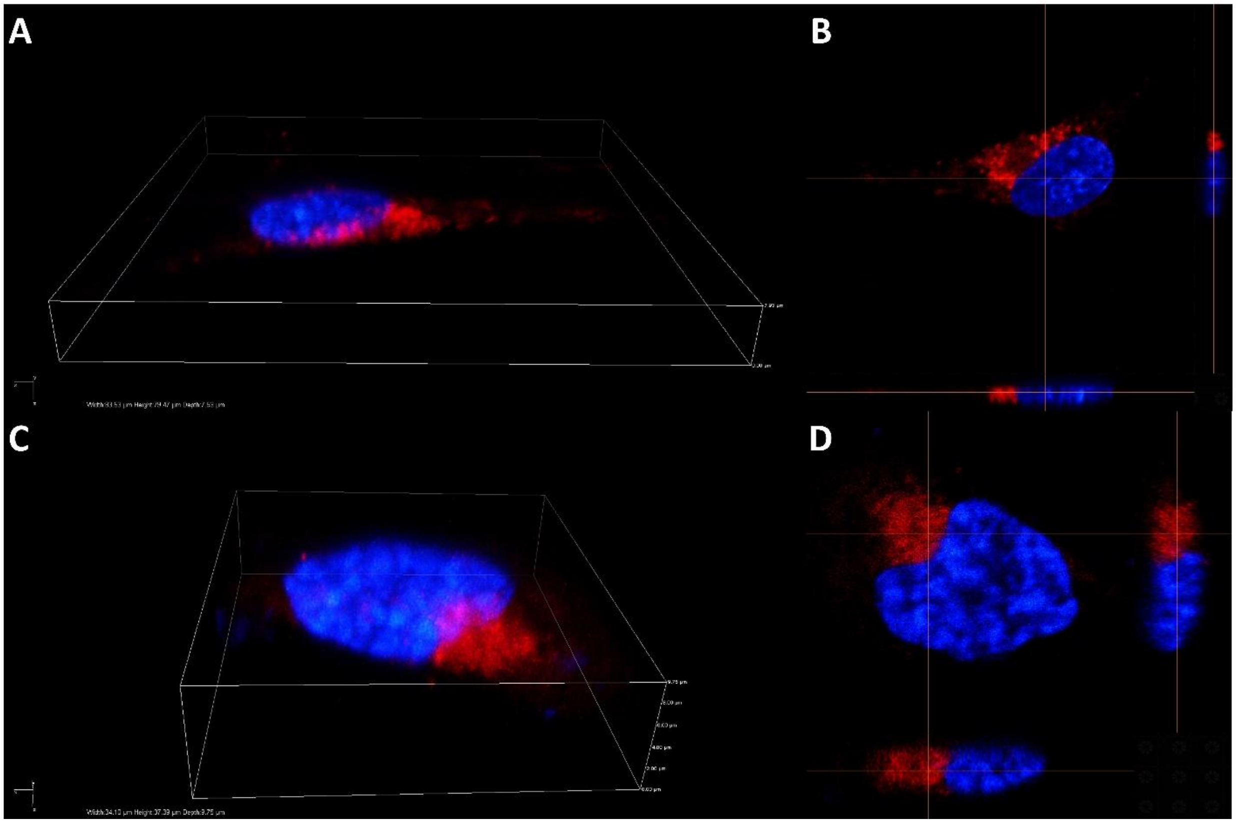

Figure S4. 3D reconstruction of HSFs (A) and HaCaTs (C) incubated with NR-loaded CS-C18_E_100 obtained using confocal scanning laser microscopy (CLSM). Cross-sectional images of HSFs (B) and HaCaTs (D) incubated with CS-C18_E_100. The nuclei were stained with Hoechst 33342 (blue fluorescence).

Acknowledgments

The project was financed by the National Science Centre, Poland (grant no. 2019/35/B/ST5/02147).
